# Dynamics of soil organic carbon under wheat cultivation in saline soil of the Yellow River Delta

**DOI:** 10.3389/fpls.2026.1855435

**Published:** 2026-07-31

**Authors:** Jia Dong, Minghui Li, Jianing Liu, Quanquan Liu, Deyong Zhao, Jikun Xu, Yunpeng Liu, Yan Wang, Long Mao, Jianlin Wang, Shuaipeng Zhao

**Affiliations:** 1 College of Biological and Pharmaceutical Engineering, Shandong University of Aeronautics, Binzhou, China; 2 Dongying Natural Resources and Planning Bureau, Dongying, China; 3 Shandong Provincal Engineering and Technology Research Center for Wild Plant Resources Development and Application of Yellow River Delta, Shandong University of Aeronautics, Binzhou, China; 4 Institute of Crop Sciences, Chinese Academy of Agricultural Sciences, Beijing, China; 5 Institute of Genetics and Developmental Biology, Chinese Academy of Sciences, Beijing, China

**Keywords:** organic carbon active components, random forest model, redundancy analysis, salinate field, soil environmental factors

## Abstract

The stability of soil organic carbon (SOC) in the saline-alkali farmlands of the Yellow River Delta is influenced by both salt stress and planting patterns. Understanding how these factors jointly regulate soil carbon pools and their active components is crucial for analyzing carbon cycling in saline-alkali lands and optimizing soil improvement strategies. This study focuses on coastal saline-alkali farmlands in the Yellow River Delta, comparing a control plot of untreated bare land with a treatment plot where wheat is cultivated following rice. Utilizing the natural salt gradient, we systematically analyzed the variations in soil water-salt environment, nutrients, enzyme activities, SOC and its active components, during the jointing, flowering, and maturity stages. Through correlation analysis, random forest, redundancy analysis, and partial least squares path modeling, we quantified how soil environmental factors regulate organic carbon components.The findings indicate that wheat cultivation significantly alters the soil water-salt and nutrient patterns in saline-alkali lands, generally enhancing soil enzyme activities. The impact of wheat cultivation on the carbon pool is modulated by salt intensity. In low-salt environments, the toxic effects of salt ions on microorganisms are mitigated, promoting the accumulation of active organic carbon components. Conversely, moderate to high salt stress significantly inhibits soil carbon turnover, with total organic carbon in the surface layer declining under these conditions. Regarding driving mechanisms, sucrase activity emerged as the key environmental factor influencing SOC changes in wheat cultivation systems. In contrast, cellulase activity and available phosphorus content were more influential in explaining organic carbon variation in the bare land system, highlighting distinct driving mechanisms of carbon pools between the two systems.Compared to the control, wheat cultivation notably enhanced the explanatory power of soil environmental factors for variations in active organic carbon components, reconstructing the regulatory network of the soil carbon cycle in saline-alkali farmlands. This study elucidates the differential and stage-specific regulatory mechanisms of wheat cultivation and salt gradients on the organic carbon pool in coastal saline-alkali lands, offering a theoretical foundation for the improvement and fertilization of saline-alkali lands and the maintenance of soil carbon pools in the Yellow River Delta.

## 1 Introduction

The Yellow River Delta, situated in northeastern Shandong Province, exemplifies a coastal saline-alkali region formed by sediment deposition at the Yellow River’s estuary (Zuo et al., 2023). Due to unique hydrogeological conditions, soil salinization has persisted here, significantly hindering sustainable agricultural development (Guo et al., 2021; Liu et al., 2023). Traditionally, this area, a vital reserve of cultivated land and a grain-producing region in Shandong Province, relied on a single-season planting model (Liu et al., 2025). Recently, however, a rice-wheat rotation model has been introduced (Wang et al., 2022) This method, which alternates between dry and wet farming, enhances soil’s physical and chemical properties and regulates the movement of water, soil, and salt (Ren et al., 1994). It offers a novel approach to improving saline-alkali farmland and boosting the soil carbon pool. Consequently, systematically comparing the effects of single-season rice planting with rice-wheat rotation on the soil carbon pool is crucial. Such comparisons aid in optimizing soil carbon management in saline-alkali lands, thereby enhancing land productivity and supporting sustainable agricultural development in these coastal areas.

Soil organic carbon (SOC) serves as a crucial indicator of soil fertility in saline-alkali farmland, contributing to the stability of soil structure and supporting ecological functions (Xv and Wang, 2017; Mi et al., 2026). The processes of SOC sequestration and turnover are vital for enhancing the carbon sink potential of regional farmland and achieving the “dual carbon” goal (Xie, 2017; Wang et al., 2025). Unlike ordinary farmland, SOC dynamics in coastal saline-alkali land are heavily influenced by soil salinity. Salt stress impacts the accumulation and transformation of the soil carbon pool through various pathways (Rath and Rousk, 2015).High salinity environments hinder crop root growth, reduce biomass input both above and below ground (Datta et al., 2019), and limit the supply of exogenous organic carbon. This environment also suppresses the activity of soil microbial communities, such as bacteria and fungi (Rath and Rousk, 2015), thereby decreasing the decomposition and transformation rates of SOC and significantly weakening soil carbon turnover efficiency (Li et al., 2013; Yang et al., 2021). Conversely, moderate salinity can enhance the formation of stable organic-inorganic complexes by promoting the interaction between soil minerals and organic matter (Xu et al., 2026). These complexes provide physical protection to SOC (Xu et al., 2025) and reduce organic carbon loss through mineralization.This intricate bidirectional regulation results in distinct SOC accumulation patterns in saline-alkali farmland compared to conventional farmland, making the evolution of the carbon pool in saline-alkali areas unique and complex.

Soil active organic carbon components are highly sensitive to environmental changes and tillage practices, making them effective indicators of dynamic changes in the saline-alkali soil carbon pool (Wang, 2020; Balontayová et al., 2025) These components are crucial for understanding the mechanisms of soil organic carbon (SOC) turnover. Dissolved organic carbon (DOC), for instance, is particularly influenced by soil water and salt transport, exhibiting significant fluctuations during the reclamation of saline-alkali land (Dong et al., 2022; Yu et al., 2023) Readily oxidizable organic carbon (ROC) reflects the short-term mineralization potential of SOC and is notably sensitive to variations in salt gradients (Li et al., 2024; Xu et al., 2024). Microbial biomass carbon (MBC) indicates the metabolic activity of soil microorganisms, which diminishes under high salt stress, leading to reduced MBC content and subsequently inhibiting soil carbon transformation and renewal processes (Wang et al., 2022; Li S. et al., 2023).While existing research has extensively examined the impact of conventional farmland tillage patterns on SOC and its active components, demonstrating that appropriate crop rotation can significantly enhance soil carbon sequestration capacity (Ma et al., 2025), there remains a gap in understanding. Specifically, there is a need for a systematic exploration of how different active carbon components respond to tillage practices and salt gradients, particularly in the unique water-salt environment of coastal saline-alkali land.

There is a substantial body of domestic and international research on farmland SOC, yet significant gaps and shortcomings persist. Primarily, most studies concentrate on the carbon cycle patterns of non-saline-alkaline cultivated land, leaving a notable lack of systematic research on SOC dynamics and its active components within the unique salt-gradient environment and rice-wheat rotation system of the Yellow River Delta’s coastal saline-alkaline land (Liu et al., 2021). Furthermore, research often emphasizes changes in overall SOC storage (Liu et al., 2024; He et al., 2026)), neglecting the distinct responses of various active carbon components. This oversight hampers the understanding of the nuanced mechanisms governing carbon pool turnover in saline-alkaline soils. Additionally, the role of winter wheat cultivation—the sole key dry-farming crop season in the rice-wheat rotation system—in regulating the carbon pool of saline-alkaline soils remains unclear. The identification of key environmental factors driving SOC accumulation and component transformation across different salt gradients is lacking, severely limiting insights into the carbon cycle mechanisms in saline-alkaline farmland and hindering the optimized implementation of the regional rice-wheat rotation model.

This study addresses the research gaps by examining saline-alkaline soils with varying salinity levels (1–7‰) in the Yellow River Delta, previously cultivated with rice. We implemented wheat planting treatments alongside control experiments to systematically track changes in soil organic carbon (SOC), its active components, and key soil environmental indicators. Utilizing redundancy analysis (RDA) and the random forest model, we identified the primary factors influencing SOC accumulation and the transformation of its components in saline-alkaline soils across different salinity gradients. Additionally, we clarified how winter wheat planting affects the dynamics of the soil carbon pool in these coastal lands. Our objective is to uncover the evolution patterns of SOC and its active components within the rice-wheat rotation system and to elucidate the soil carbon pool’s response to the combined effects of salinity and tillage. This research aims to provide a scientific foundation and practical guidance for optimizing tillage systems, conserving soil fertility, and enhancing carbon sequestration potential in the saline-alkaline farmlands of the Yellow River Delta.

## 2 Materials and methods

### 2.1 Study region

In 2024, a fixed-point field experiment was conducted in Kenli District, Dongying City, Shandong Province (37°42′N, 118°53′E). Situated at an altitude of 2 meters, the area experiences a warm-temperate continental monsoon climate. The annual average temperature ranges from 12.3 to 13.8 °C, with average annual precipitation between 534 and 547 mm. In contrast, the average annual water surface evaporation reaches 1500 mm, significantly exceeding precipitation. This disparity is a key climatic factor contributing to the secondary salinization of coastal soils.

The experimental area’s soil, formed by the sedimentation of the Yellow River’s estuarine deposits, is classified between sandy loam and light loam. The study uses the soil salinity classification standards for coastal saline-alkali farmland in the Yellow River Delta, as well as similar regional studies (Guan et al., 2001; Xu and Li, 2022). The soil is categorized into three salinity gradients: low-salt (1-3‰), medium-salt (3-5‰), and high-salt (5-7‰).

In this experiment, gradients were categorized according to the background salt content measured in the plots. The low-salt treatment had a total soil salt content of 2.1‰, corresponding to an EC of 1.428 mS/cm in a 1:5 soil-water extract. The medium-salt treatment exhibited a salt content of 4.3‰ with an EC of 2.924 mS/cm, while the high-salt treatment had a salt content of 6.1‰ and an EC of 4.148 mS/cm.Before sowing, mixed soil samples from the 0–20 cm plow layer were analyzed to establish the initial soil fertility background. The samples, representing the basic soil from blank plots across the three salinity gradients, were measured for total nitrogen (35.34 mg/kg), available phosphorus (28.51 mg/kg), available potassium (130.81 mg/kg), organic matter (14.70 g/kg), and pH (8.58). Three replicates were set for each gradient to ensure accuracy.

### 2.2 Experiment design and sample treatment

The experiment employed a two-factor completely randomized design, focusing on salinity gradient and planting treatment. Initiated in late October 2024, the study utilized the winter wheat variety Nongda 753, sown manually by dibbling. Six treatment groups were established: low-salinity wheat (1 - 3‰, LS-W), medium-salinity wheat (3 - 5‰, MS-W), and high-salinity wheat (5 - 7‰, HS-W). Corresponding bare soil controls were also included: low-salinity control (LS-CK), medium-salinity control (MS-CK), and high-salinity control (HS-CK). All plots previously grew rice, sharing a consistent tillage history and baseline water and fertilizer conditions. Each treatment group covered an area of 667 m², with three biological replicates per treatment. A 1.5 m isolation buffer separated plots to prevent lateral interference from soil salinity, water, and fertilizer. Wheat plots were managed according to local standards for rice-wheat rotation, ensuring uniform tillage and fertilization. In contrast, the control plots (CK) remained unsown, with no external fertilization or irrigation, and weeds were manually removed to maintain bare soil. The wheat harvest occurred uniformly across all fields in early June 2025.

Soil samples were collected at three crucial growth stages of winter wheat: the jointing, flowering, and maturity stages. Sampling was conducted in the 0–20 cm plow layer of the farmland using a 5 cm diameter soil auger. In each experimental plot, the three-point diagonal sampling method was employed, and samples from the same plot were combined into a single composite sample. After collection, the samples were divided into three parts on-site. The first portion, weighing 40–50 g, was placed in an aluminum box to measure soil water content. The second portion was stored in a sterile, self-sealing bag at 4°C in the dark for assessing soil microbial biomass carbon. The remaining soil was air-dried indoors in a well-ventilated area, with crop roots, stones, and other impurities removed. This portion was then passed through a 20 mm nylon sieve and ground for subsequent analysis of other soil physical and chemical indicators and organic carbon fractions. All soil sample analyses were conducted with three technical replicates for accuracy.

### 2.3 Measurement of soil environmental factor index

The physical and chemical properties of soil, along with the activities of soil hydrolases, were assessed using standard soil agrochemical detection methods. The specific procedures are as follows (Guan, 1986; Lu, 1986; Bao, 2000) Soil moisture content (SMC) was determined by the constant-temperature drying and weighing method at 105 °C. Soil electrical conductivity (EC) was measured using a portable electrode method after extraction with a 5:1 water-to-soil ratio. Available phosphorus (AP) was assessed via the sodium bicarbonate extraction-molybdenum-antimony anti-colorimetric method. Total nitrogen (TN) was measured using the semi-micro Kjeldahl method. Available potassium (AK) was determined through the ammonium acetate extraction-flame photometry method. The activities of soil sucrase (SSA), amylase (SAA), and cellulase (SCA) were consistently measured using the 3, 5-dinitrosalicylic acid colorimetric method (DNS method).

### 2.4 Measurement of SOC and active components in soil

Soil organic carbon (SOC) was determined using the combustion oxidation method with a Total Organic Carbon (TOC) analyzer. A 0.5 g soil sample was treated with 2 mol/L hydrochloric acid (HCl) until no effervescence was observed, then allowed to stand for 12 hours. The sample was subsequently washed with distilled water until neutral, dried at 60 °C, and ground. Following this preparation, 10–20 mg of the processed soil sample was placed into the TOC analyzer for high-temperature combustion. The instrument automatically detected the concentration of CO_2_, and the SOC content was calculated based on a standard curve using the formula (Ren et al., 2017):


$$\;SOC\;(g/kg)\;=\;(A\;-\;A_{0}\;-\;a)\;/\;(b\;\times \;m_{1})\;\times \;100\;$$

where A is the infrared absorption response value of the test sample, A_0_ is the response value of the blank sample, a is the intercept of the calibration curve, b is the slope of the calibration curve, and m_1_ is the mass of dry matter in the test sample (g).

Microbial biomass carbon (MBC) was quantified using the chloroform fumigation-extraction method. The procedure involved placing soil samples in a vacuum desiccator containing a small amount of chloroform. The desiccator was evacuated until chloroform boiled continuously for 2 minutes. Following this, the valve was closed, and fumigation was conducted in the dark at 25°Cfor 24 hours. After fumigation, the desiccator valve was opened, and a vacuum-nitrogen purge cycle was repeated three times to completely remove chloroform. Both fumigated and non-fumigated soil samples were extracted with 80 mL of 0.5 mol/L K_2_SO_4_ solution (with a soil-to-solution ratio of 1:4) by shaking at 25 °C for 30 minutes, followed by centrifugation at 4000 r/min for 15 minutes. The supernatant was then filtered through a 0.45 μm membrane, and the filtrate was stored at 4°C until analysis. Finally, the organic carbon concentration (mg/L) of the extract was measured using a TOC analyzer. The calculation formula is as follows (Ren et al., 2017):


MBC (mg/kg)=[(C1−C2)×V×kEC]/m×1000

where C1 represents the organic carbon concentration of the extract from the fumigated sample (mg/L); C2 denotes the organic carbon concentration of the extract from the non-fumigated sample (mg/L); V is the volume of the extract (0.08 L); kEC is the conversion coefficient, taken as 0.45; and m is the oven-dried mass of the soil sample (kg).

Soil dissolved organic carbon (DOC) was determined using the water extraction method, followed by analysis with a Total Organic Carbon (TOC) analyzer. A fresh soil sample weighing 10.0 g, which had been passed through a 2-mm sieve, was placed into a 100 mL centrifuge tube. Deionized water was added at a soil-to-water ratio of 1:5 (50 mL), and the mixture was shaken at a constant temperature of 25 °C for 30 minutes at 200 revolutions per minute (r/min). Subsequently, the mixture was centrifuged at 4000 r/min for 15 minutes. The supernatant was then filtered through a 0.45 μm aqueous filter membrane, and the resulting filtrate was injected into the TOC analyzer. Using deionized water as a blank, the DOC concentration (mg/L) was directly measured, and the content was calculated using the following formula (Yang et al., 2023):


DOC (mg/kg)=(C×V)/m

Where C is the measured DOC concentration in the extract (mg/L); V is the total volume of the extract (L); m is the mass of the air-dried soil sample (kg).

Soil readily oxidizable carbon (ROC) was quantified using the potassium permanganate oxidation colorimetric method. A 1 g air-dried soil sample was placed in a 50 mL centrifuge tube, followed by the addition of 5 mL of 0.0333 mol/L KMnO_4_ solution and 1 mL of 0.1 mol/L H_2_SO_4_ solution. Subsequently, 0.2 mL of the supernatant was transferred to a 50 mL volumetric flask and diluted to the mark. The absorbance of the diluted solution was measured at a wavelength of 565 nm using a spectrophotometer. A standard curve was constructed using glucose solutions, and the ROC content was calculated by comparing the sample absorbance with this standard curve. The calculation formula is given as follows (Blair et al., 1995):


ROC (g/kg)=V×25×250×9/( m×1000 )

where V represents the change in potassium permanganate concentration; 25 and 250 denote the volume (mL) of potassium permanganate used and the dilution factor, respectively; 9 indicates the amount of carbon (mg) consumed corresponding to a 1 mmol/L change in KMnO_4_ concentration; and m is the soil mass (g).

### 2.5 Data processing and analytics

Excel 2021 facilitated the organization of raw data, outlier elimination, and basic data preprocessing. For statistical analysis, SPSS 27.0 (IBM, Armonk, NY, USA) was employed. A one-way analysis of variance was performed to examine soil environmental factors, soil organic carbon (SOC), and its active components across different treatments during the same growth period. To assess differences between the wheat treatment group and the blank control group, an independent samples t-test was conducted based on planting treatment groupings. Additionally, a Pearson correlation heatmap was created using Origin 2024 software to explore the linear relationships between soil environmental factors and active organic carbon components, including microbial biomass carbon (MBC), dissolved organic carbon (DOC), and readily oxidizable carbon (ROC).

Redundancy analysis (RDA) was conducted using Canoco 4.5 software. Prior to analysis, all environmental factors and organic carbon component data were standardized through a log(x + 1) transformation. Variables exhibiting multicollinearity were eliminated using the variance inflation factor (VIF). The constrained ordination included soil organic carbon (SOC), microbial biomass carbon (MBC), dissolved organic carbon (DOC), and readily oxidizable carbon (ROC) as response variables, while soil physical and chemical properties and enzyme activity factors served as explanatory variables. The variance explanation rates for each ordination axis and the contributions of individual environmental factors were statistically analyzed to determine how environmental factors explain the spatial variation of soil organic carbon components.

The random forest regression model was implemented using R 4.2.3, with soil organic carbon (SOC) as the response variable and seven soil environmental factors as explanatory variables. The model’s core parameters included 1, 000 decision trees (ntree = 1000) and two variables randomly sampled at each split (mtry = 2), which is the default for regression models when the total number of independent variables is seven. Internal model validation was conducted using the built-in out-of-bag (OOB) error, without employing external k-fold cross-validation. The model’s evaluation relied on the %IncMSE (percentage increase in mean squared error) to assess the importance of environmental factors. To determine the significance of these factors, a Monte Carlo permutation test with 299 repetitions (nrep = 299) was performed, identifying key environmental drivers of SOC accumulation at significance levels of *P<0.05、**P<0.01.

The PLS-PM model was developed using the plspm package in R to examine the causal regulatory relationships among soil water and salt, nutrients, extracellular enzymes, active carbon components, and total SOC within the saline-alkali rice-wheat rotation system. The model incorporated five latent variables: soil water and salt (EC, SMC), soil nutrients (AP, AK, SSA, SAA, SCA), extracellular enzyme activity (ROC), active organic carbon (DOC, MBC), and total organic carbon (SOC). Initially, all observed indicators were standardized using the Z-score method, and extreme outliers were excluded based on the 3σ rule. Indicators with weights less than 0.4 were removed during the preliminary screening of the measurement model. The model’s predetermined causal pathway was structured as follows: soil salinity → soil nutrients → soil enzyme activity → active carbon → soil organic carbon pool. To enhance the model’s explanatory power and address index covariance conflicts due to salinity’s spatial heterogeneity, further purification of the measurement model was conducted. Indicators with external loads below 0.7 within the same latent variable and those with conflicting correlation coefficients were eliminated, along with structural paths lacking statistical significance (P > 0.05). Both direct and indirect regulatory effects of variables at each level were simultaneously output.In this study, a multi-index joint evaluation system was utilized to comprehensively assess the model’s reliability, rather than relying solely on a single SRMR threshold for evaluating the fit. According to classic PLS-SEM specifications, a GOF of 0.36 or higher is considered a good fit for field soil datasets with spatial heterogeneity. For highly heterogeneous saline-alkali farmlands, a GOF between 0.30 and 0.50 is deemed acceptable (Tenenhaus et al., 2005; Wetzels et al., 2009; Tong et al., 2024). The strict SRMR standard of less than 0.08 is applicable only to homogeneous, low-noise indoor experimental or survey data. For field ecological data characterized by micro-scale salt patches and conflicting correlations among latent variable indicators, the academic community advises a comprehensive evaluation of the model’s rationality by considering GOF, index load, path significance, and the R^2^ of endogenous latent variables (Nguyen, 2025). In this study, path significance, standardized path coefficients, and the R^2^ of endogenous variables were also pivotal in elucidating the mechanisms of soil carbon cycling.

To further validate the structural soundness of the theoretical model and the stability of parameter estimates, this study incorporates two standard robustness testing methods commonly used in the field of Partial Least Squares Structural Equation Modeling (PLS-SEM). Initially, three sets of alternative models—baseline theoretical model, pure cascade chain model, and simplified model—were constructed for comparative analysis. This approach assesses the necessity of direct paths across levels and the theoretical retention value of non-significant paths. Subsequently, a bootstrap resampling method with 1, 000 iterations was employed to calculate the 95% percentile confidence intervals for all path coefficients. This step ensures that the estimation results remain stable, unaffected by sampling fluctuations and outliers. Detailed results of the robustness tests are provided in the **Supplementary Materials**.

## 3 Results

### 3.1 Changes in soil environmental parameters of saline-alkali soils under different treatment conditions

**Figure 1:**
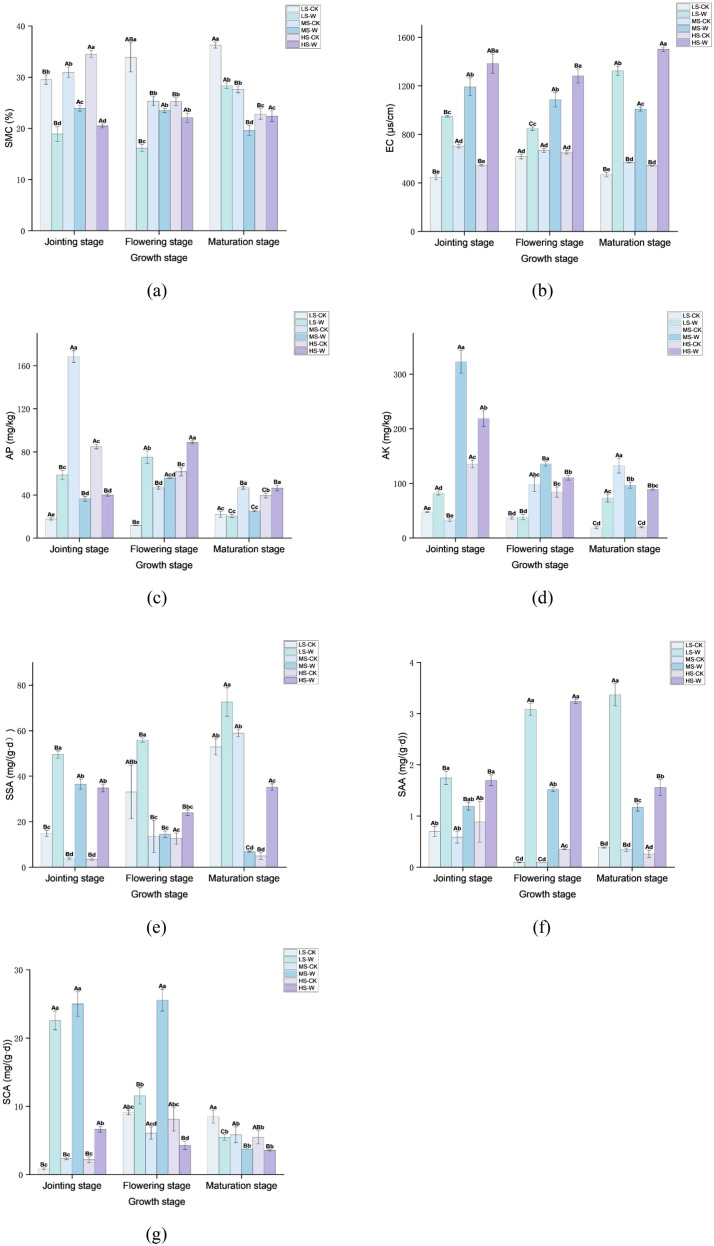
Variation trends of soil environmental parameters under different treatment conditions are illustrated. Uppercase letters denote significant differences between various time periods within the same treatment, while lowercase letters indicate significant differences among different treatments during the same period (p < 0.05). The parameters represented include **(a)** SMC; **(b)** EC; **(c)** AP content; **(d)** AK content; **(e)**SSA; **(f)** SAA; **(g)** SCA.

**Figure 1** illustrates the dynamic changes in soil environmental parameters under various treatment conditions. Throughout the growth period, the SMC in the wheat planting group remained lower than in the blank control group. Notably, the SMC in the LS-W exhibited a continuous increase as the growth process progressed. In contrast, the soil EC in the wheat group was generally higher than in the control group, with the HS-W maintaining the highest EC levels across the three sampling periods.At the crop maturity stage, the levels of AP and AK, generally reached their lowest points. During the jointing stage, the AP content in the MS-CK was significantly higher than in all other treatments. Similarly, the AK content in the wheat group was significantly higher than in the control group during the jointing stage, although the MS-CK exhibited the highest AK content at maturity among all groups.The SSA increased consistently across all treatments throughout the growth period. The SSA in the wheat group was significantly higher than in the control group, with the LS-W showing a notably higher SSA than other treatments during all three periods. SAA was generally elevated in the wheat planting soil, with the LS-W demonstrating the highest SAA among all groups throughout the entire process. Meanwhile, SCA in the wheat group surpassed that of the control group at the jointing stage, but the control group exhibited higher activity at the maturity stage.

### 3.2 Dynamics of SOC and its active components under various treatments and salinity-alkalinity gradients

**Figure 2:**
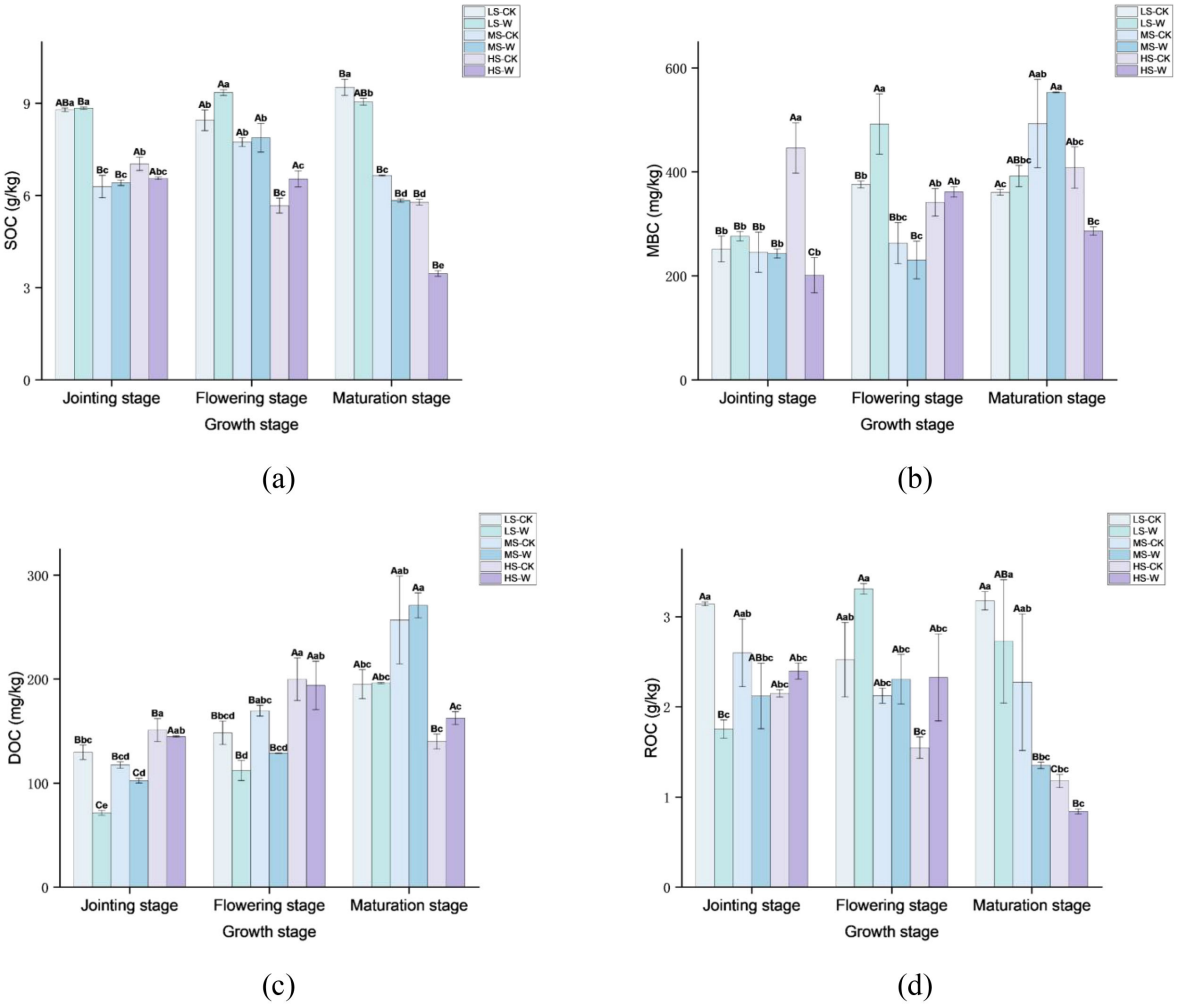
Dynamic changes in soil organic carbon and its active components under various treatment conditions were observed. Different uppercase letters denote significant differences between time periods within the same treatment, while different lowercase letters indicate significant differences among various treatments during the same period (p < 0.05). The components analyzed include: **(a)** SOC content; **(b)** MBC content; **(c)** DOC content; **(d)** ROC content.

**Figure 2** illustrates the dynamic changes in SOC and its active components throughout the growth period under various treatments. At the jointing stage, SOC levels showed no significant difference between the wheat-planting group and the control group. However, SOC content was notably higher in the low-salt treatment compared to other groups. By the maturity stage, SOC in the wheat group was generally lower than in the control group with equivalent salinity, and this disparity increased with rising salinity. Specifically, SOC decreased by 4.94%, 12.23%, and 40.17% in the low-salt, medium-salt, and high-salt groups, respectively. DOC in the low-salt and medium-salt wheat treatments increased steadily as growth progressed. At maturity, the DOC content in the MS-W was the highest among all groups. During the jointing and flowering stages, DOC in the wheat group was lower than in the control group with the same salinity. However, this trend reversed at maturity, with DOC in the wheat group surpassing that of the control group. MBC increased progressively with wheat growth. At the jointing stage, MBC in the HS-CK was significantly higher than in other treatments. Conversely, at the flowering stage, the LS-W exhibited significantly higher MBC content than other treatments. ROC remained relatively stable in the low-salt and medium-salt control groups. Overall, ROC in the wheat-planting group was lower than in the control group with the same salinity, except during the flowering stage, where ROC in the wheat group exceeded that of the corresponding blank control.

### 3.3 Relationship between soil environmental factors and soil organic carbon with its active components

**Figure 3:**
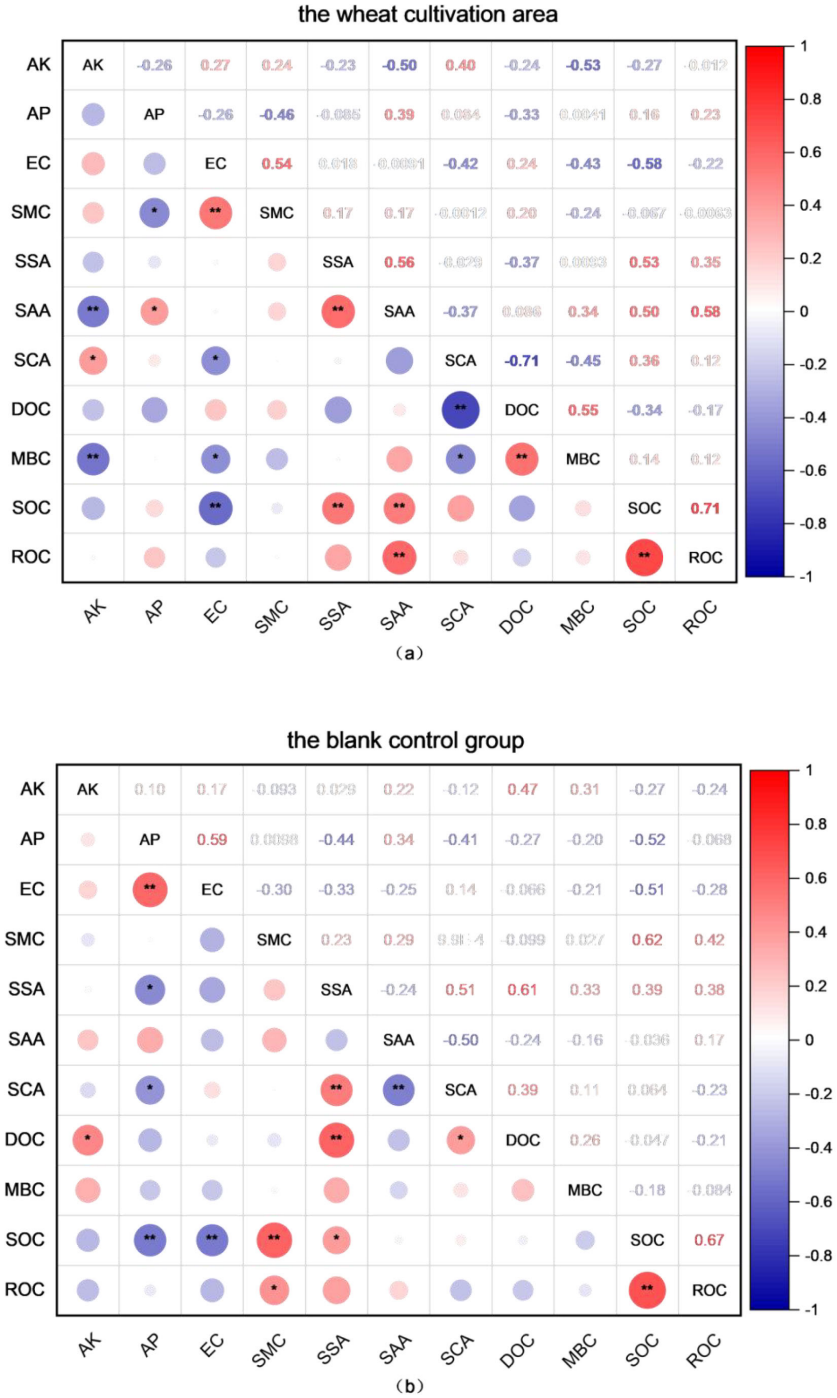
Correlation analysis of soil physicochemical properties, soil organic carbon, and its active component levels under wheat cultivation and control treatments: **(a)** the wheat cultivation area; **(b)** the blank control group. Red circles indicate positive correlations, whereas blue circles represent negative correlations. The size of the circles reflects the strength of the association, and the numbers denote the correlation coefficients. The symbols (*) and asterisks (**) indicate significant correlations at the 0.05 and 0.01 probability levels, respectively.

**Figure 3** presents a Pearson correlation heatmap illustrating the relationships between soil environmental indicators and organic carbon, along with its active components. In the wheat planting group, there is a notable positive correlation between SAA and SOC with ROC. Additionally, both SSA and SAA show a significant positive correlation with SOC. Conversely, EC exhibits a significant negative correlation with ROC. DOC is positively correlated with MBC, while AK, EC, and SCA are negatively correlated with MBC; SCA also shows a negative correlation with DOC.In the blank control group, SOC demonstrates a significant positive correlation with both SMC and ROC. Similarly, SMC and SSA are positively correlated with SOC. However, AP and EC are negatively correlated with SOC. Furthermore, AK, SSA, and SCA show significant positive correlations with DOC.

### 3.4 Effects of soil environmental factors on soil organic carbon content

**Figure 4:**
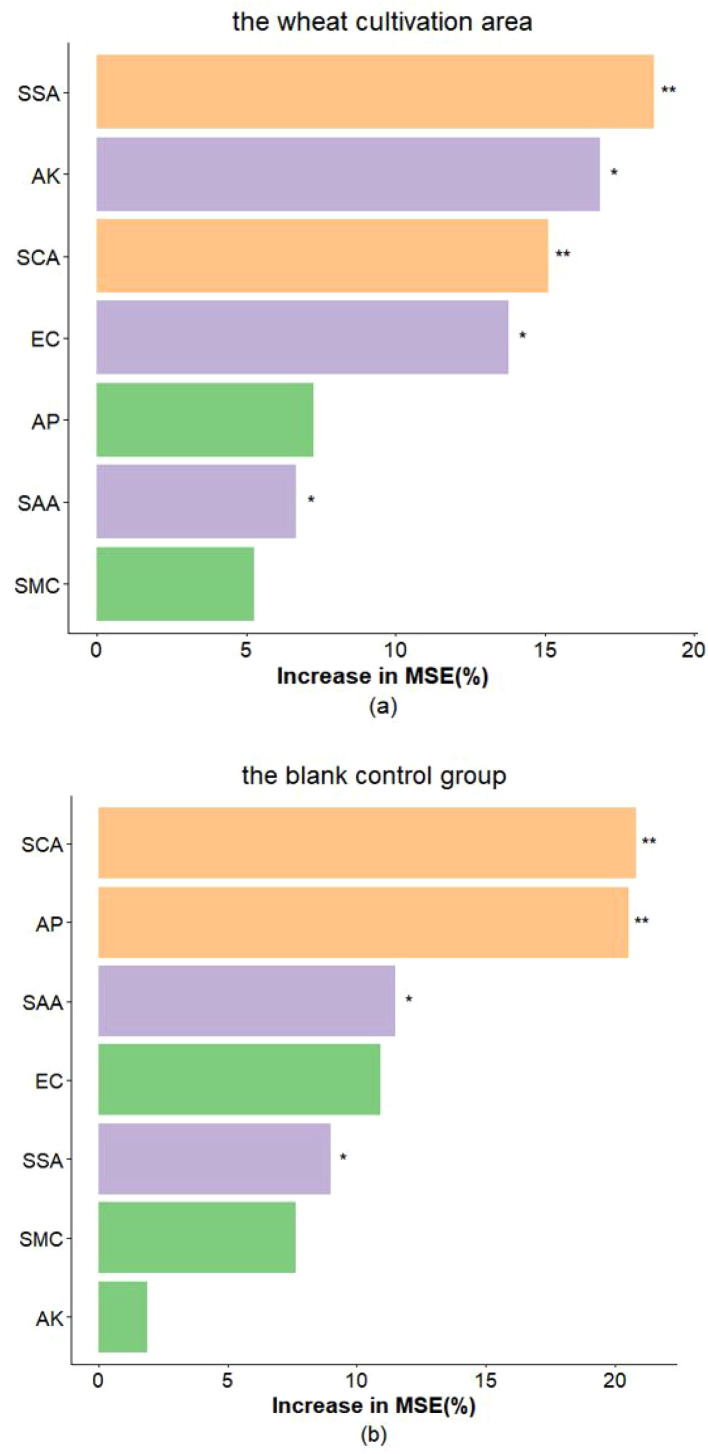
Comparison of the relative importance of soil environmental factors to soil organic carbon (SOC) content under the two treatment patterns: **(a)** the wheat cultivation area and **(b)** the blank control group. The symbols (*) and (**) denote significant and highly significant correlations, respectively, between SOC and the corresponding factors.

This study employed a random forest model to assess the relative importance of various soil environmental factors on SOC. The findings, depicted in **Figure 4**, reveal that in the wheat planting group, SSA emerged as the most influential factor, contributing 18.65% to SOC. Following SSA, AK, SCA, and EC contributed 16.85%, 15.11%, and 13.79%, respectively. Conversely, AP, SAA, and SMC had relatively minor impacts on SOC. In contrast, within the blank control group, SCA and AP were identified as the primary drivers, with contributions of 20.84% and 20.54%, respectively. The contributions of SAA, EC, and SSA to SOC were 11.53%, 10.92%, and 8.99%, respectively, indicating that other environmental factors had limited explanatory power regarding SOC.

### 3.5 Redundancy analysis of soil environmental factors and active components of soil organic carbon

**Figure 5:**
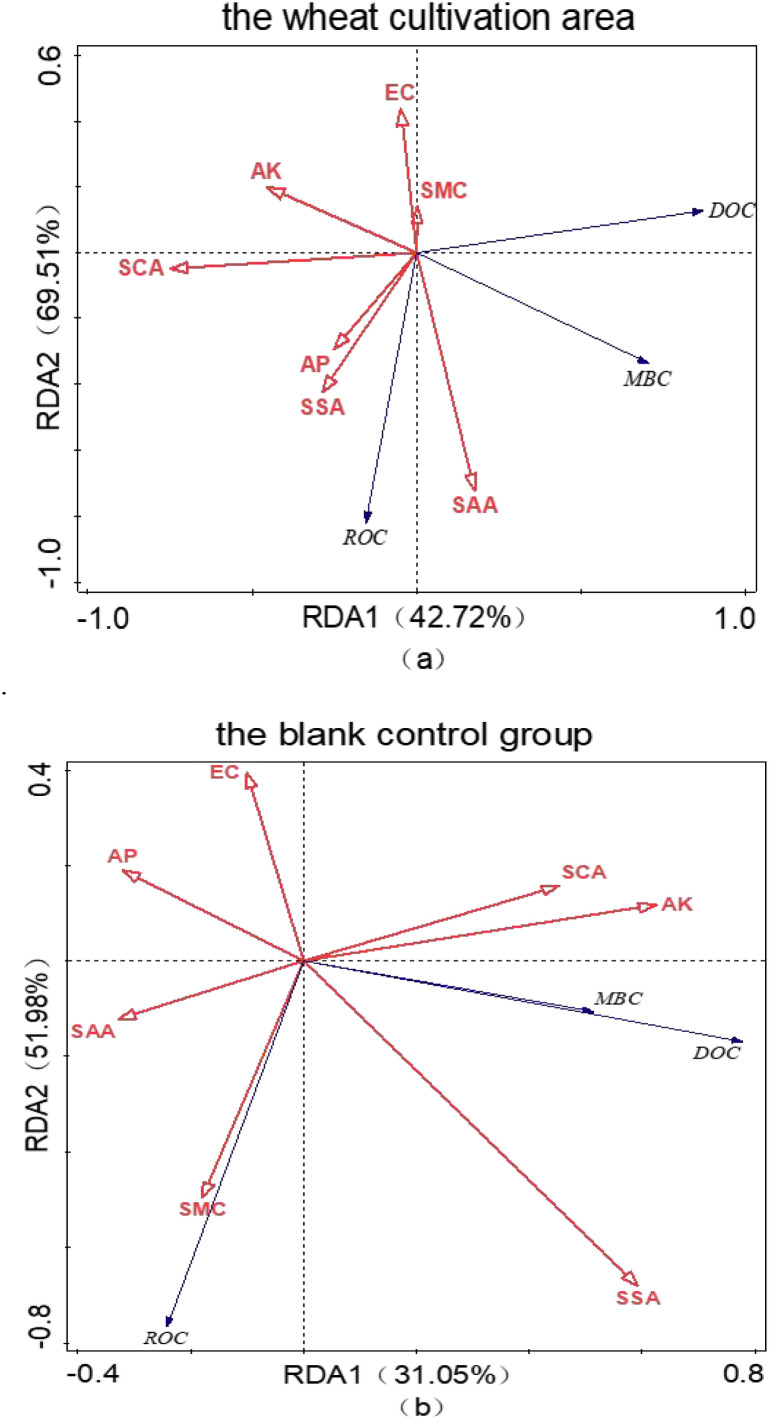
Redundancy analysis of soil environmental parameters and active organic carbon components under the wheat cultivation pattern: **(a)** the wheat cultivation area; **(b)** the blank control group. The red lines represent soil physicochemical properties, while the blue lines denote active components of soil organic carbon. An angle exceeding 90° between two lines indicates a negative correlation between the two variables; an angle of 90° suggests no correlation; and an angle less than 90° reflects a positive correlation. The perpendicular distance from the arrowhead of one line to another line indicates the strength of their association. RDA1 and RDA2 correspond to the explanatory proportions of the first and second principal components, respectively.

**Figure 5** illustrates the redundancy analysis of soil environmental parameters and active fractions of soil organic carbon under two different planting patterns. In the wheat planting treatment, soil environmental parameters accounted for 77.5% of the variation in active organic carbon fractions. The first and second ordination axes contributed 42.72% and 69.51%, respectively. Notably, SCA (24.3%, F = 8, p = 0.002), SAA (17.6%, F = 7.3, p = 0.002), EC (15.0%, F = 8, p = 0.002), SMC (7.5%, F = 4.6, p = 0.008), and AP (7.6%, F = 6.1, p = 0.01) had significant explanatory contributions to the active organic carbon fractions, whereas SSA and AK did not show significant effects.In contrast, within the blank control group, soil environmental parameters explained 54.2% of the variation in active organic carbon fractions. The contribution rates for the first and second ordination axes were 31.05% and 51.98%, respectively. SSA (20.7%, F = 6.5, p = 0.002), AK (11.9%, F = 4.2, p = 0.004), and SCA (10.7%, F = 4.3, p = 0.018) demonstrated significantly higher explanatory contributions compared to other environmental factors, while SMC, EC, SAA, and AP did not have significant explanatory effects.

### 3.6 Interaction mechanism between soil environment, SOC, and active components of SOC

**Figure 6:**
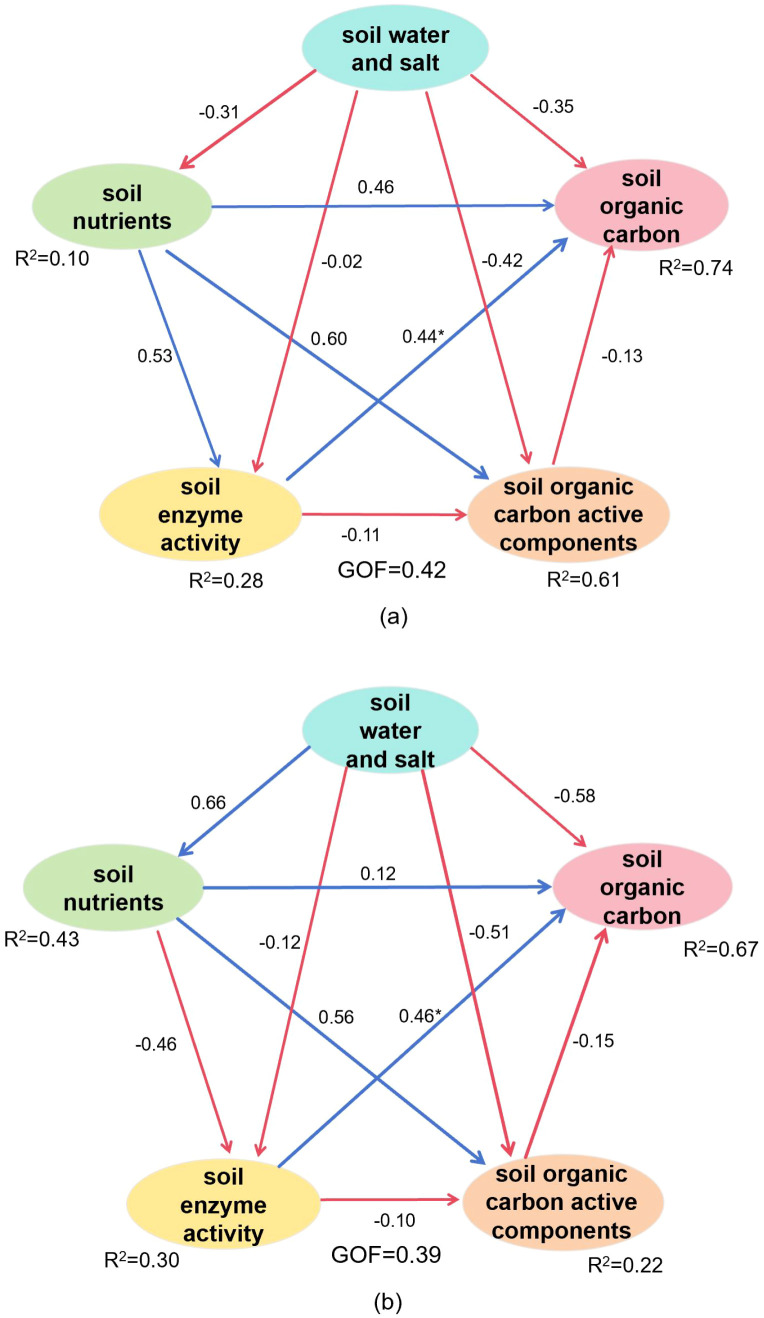
The partial least squares path model illustrates the relationship between soil environmental factors, soil organic carbon, and active organic carbon components under various treatments: **(a)** the wheat cultivation area; **(b)** the blank control group. The goodness-of-fit (GOF) indicates how well the model fits the data. Interpretation thresholds are as follows: GOF ≥ 0.36 indicates good fit, 0.25–0.36 is moderate, and 0.10–0.25 is low (Tong et al., 2024; Fu et al., 2026).The coefficient of determination (R²) reflects the predictive power of the explanatory variables on the endogenous variables, with the starting point representing the explanatory variable and the endpoint representing the endogenous variable.The color blue signifies a positive correlation, red denotes a negative correlation, and the accompanying numbers represent the strength of these correlations. The symbols * indicate significant and highly significant effects, respectively.

**Figure 6** illustrates the comprehensive regulatory pathways linking soil water and salt conditions with nutrients, enzyme activities, active organic carbon components, and total organic carbon under two different treatments. The model employs a full-forward path structure to encompass all potential direct and indirect regulatory relationships.The model’s global goodness-of-fit (GOF) for the wheat planting group, as depicted in **Figure 6a**, was 0.42. It accounted for 10%, 28%, 61%, and 74% of the variations in soil nutrients, enzyme activities, active organic carbon components, and soil total organic carbon (SOC), respectively. Soil water and salt negatively impacted soil nutrients (path coefficient = -0.31), enzyme activities (-0.02), active organic carbon components (-0.42), and total organic carbon (-0.35); however, these effects were not statistically significant. Although soil nutrients positively influenced soil enzyme activities (0.53), active organic carbon components (0.60), and total organic carbon (0.46), these effects also lacked statistical significance. The negative impact of soil enzyme activities on active organic carbon components was insignificant (-0.11), yet it significantly promoted total organic carbon (0.44*). The regulatory effect of active organic carbon components on total organic carbon was negative and not statistically significant (-0.13).The global goodness-of-fit for the model concerning the blank bare land control group (**Figure 6b**) was 0.39. The coefficients of determination (R²) for the relationships between soil nutrients, enzyme activities, active organic carbon fractions, and total organic carbon were 43%, 30%, 22%, and 67%, respectively. While soil water and salt showed a positive but non-significant effect on soil nutrients (0.66), they exhibited negative effects on enzyme activities (-0.12), active organic carbon fractions (-0.51), and total organic carbon (-0.58), none of which were statistically significant. Soil nutrients demonstrated a negative but non-significant impact on enzyme activities (-0.46) and positive effects on active organic carbon fractions (0.56) and total organic carbon (0.12), without reaching statistical significance. Similar to the wheat planting group, soil enzyme activities significantly positively influenced total organic carbon (0.46*), though their effect on active organic carbon fractions was non-significant (-0.10). Additionally, active organic carbon fractions had a non-significant negative impact on total organic carbon (-0.15).

### 3.7 Robustness test of the PLS-PM model

This study aimed to verify the optimality of the benchmark theoretical model by comparing the fitting effects of three model specifications across two sample groups: untreated bare land and land planted with wheat (**Supplementary Table 1**).The results from both groups exhibit a consistent pattern: when all direct shortcut paths are removed, the explanatory power of the pure cascade chain model for soil organic carbon (SOC) significantly diminishes. Compared to the baseline model, the R² for SOC in the bare soil group drops by 97%, while in the wheat cultivation group, it decreases by 67%. This finding confirms the indispensable direct impact of water-salt conditions and nutrients on carbon pools, validating the structural soundness of the baseline model’s multipath framework. Although the simplified model’s explanatory power for SOC is similar to that of the baseline model, its overall goodness of fit declines, and the explanatory power for the key intermediate variable, active carbon, is severely compromised. This indicates that statistically insignificant paths still hold crucial mechanistic value and should be retained in the theoretical model to support the analysis of carbon cycle regulation mechanisms.

The results from 1000 Bootstrap resampling confirm that the core regulatory conclusions of the model exhibit strong statistical robustness (**Supplementary Table 2**). In both sample groups, the 95% confidence intervals for the key functional path “soil enzyme activity → total organic carbon” do not intersect zero. Additionally, the resampled mean values align closely with the original estimates in both direction and magnitude. This consistency indicates that the core regulatory path remains unaffected by sampling fluctuations and outliers, ensuring the stability of the estimated results. For other paths, the high spatial heterogeneity of field soils slightly diminishes the robustness of some direct effects. However, the directions of these effects remain entirely consistent with the baseline model, and no results exhibit sign reversals. Furthermore, for all non-significant paths in the original model, the confidence intervals continue to cross zero after resampling, indicating no false positive results.

In summary, the core regulatory conclusions of the model are stable and reliable.

## 4 Discussion

This study investigated the coastal saline-alkali rice-wheat rotation farmland in the Yellow River Delta, focusing on the interactions among soil water and salt, nutrients, soil enzymes, and organic carbon pools under wheat planting regulation. It examined the dynamic differentiation patterns of SOC and its active components—DOC, MBC, and ROC—in saline-alkali farmland. The research aimed to clarify how different soil environmental factors influence the carbon cycle process and to quantify the multi-factor collaborative regulation relationship. This was achieved through correlation analysis, random forest, RDA, and PLS-PM. Ultimately, the study elucidated the impact of wheat planting on carbon sequestration in saline-alkali soils.

The dynamic changes in soil water, salt, nutrients, and enzyme activities during the growth period result from the combined effects of crop root consumption, soil salinization, and microbial substrate supply. Generally, SMC in the control plots is higher than in wheat-planted plots. This difference arises not merely from water storage levels but primarily due to water consumption through crop transpiration (Pan et al., 2005). Wheat’s shallow roots actively absorb surface soil water for growth and grain filling (Zhu et al., 2026), and precipitation infiltration cannot sufficiently replenish the continuous transpiration loss. In control plots without crop cover, the absence of vegetation water consumption allows precipitation to remain in the surface soil, maintaining higher moisture levels. Additionally, soil EC increases in wheat-planted areas (Wu et al., 2024). In coastal saline-alkali soils, surface water evaporation leads to salt accumulation on the soil surface. A high salt gradient further elevates soil ion concentration. Excessive Na^+^ can impair root cell membrane permeability, reducing the crop’s ability to absorb soluble salts and exacerbating surface salt accumulation.

During the crop growth cycle, APandAK reached their lowest levels at the maturity stage. This decline resulted from peak nutrient absorption by crops and salt stress inhibiting microbial mineralization (Fageria and Oliveira, 2014; Wu, 2023). At maturity, wheat grain development demands maximum nitrogen, phosphorus, and potassium (Fageria and Oliveira, 2014), leading to substantial nutrient uptake by roots. Concurrently, high salt levels suppress soil microbial activity (Li et al., 2013), slowing the conversion of organically-bound nutrients into available forms, further depleting soil nutrient reserves.At the jointing stage, AP content in the MS-CK treatment was significantly higher than in other treatments. This was due to the absence of continuous phosphorus consumption by crops in the control plot, allowing available phosphorus from soil organic phosphorus mineralization to accumulate. In contrast, wheat roots rapidly increased phosphorus demand at this stage, continuously depleting soil AP and creating significant differences between groups.Overall, AK content in wheat-planted plots was higher than in control plots at the same stage. Low-molecular-weight organic acids secreted by wheat roots activate insoluble potassium (Wang, 2005). Additionally, K^+^ adsorbed on soil colloids competes with free Na^+^ in saline environments for desorption (Wang, 2005), collectively enhancing available potassium supply in the soil. By maturity, AK content in the medium-salt control group exceeded that in wheat-planted plots. At this point, competitive desorption of salt ions peaked, with colloids continuously releasing available potassium. In the control plot, without crop uptake, potassium accumulated, whereas wheat roots continued absorbing potassium for grain supply. Consequently, AK content in wheat-planted plots was lower than in the MS-CK treatment.

Wheat cultivation significantly boosts the activities of SSA and SAA. The root exudates from crops serve as crucial substrates that regulate the enzyme-producing capabilities of microorganisms (Arshad et al., 2022; von Haden et al., 2024). Wheat roots consistently release soluble carbohydrates and organic acids, providing a stable carbon source for soil heterotrophic microorganisms. This process stimulates the proliferation of SAA-producing microorganisms, thereby enhancing SAA activity. In contrast, fallow plots lack the continuous input of root exudates, leading to an insufficient substrate supply for microorganisms. This limitation reduces the community size and enzyme-producing potential (Li et al., 2011), resulting in overall low enzyme activity.Significant differences in SCA activity are observed at various growth stages. During the jointing stage, SCA in wheat plots surpasses that in control plots, but it falls below control levels at the maturity stage (Boudabbous et al., 2022). At the jointing stage, ample root residues and exudates stimulate the proliferation of antioxidant microorganisms (Bardelli et al., 2024). As growth progresses, easily decomposable organic substrates in the soil are gradually consumed. Concurrently, long-term salt stress diminishes microbial metabolic activity, leading to a decrease in SCA activity. In fallow plots, the slow decomposition of inert organic matter releases substrates, and stable aeration conditions favor the enrichment of salt-tolerant and low-temperature microorganisms. Consequently, SCA activity gradually recovers by the maturity stage.

The regulation of SOC turnover and its active components by salt gradients and wheat cultivation is a complex interaction (Liu et al., 2020; Aizizi et al., 2025). In low-salt environments, the toxic effects of salt ions on soil microorganisms are reduced, enhancing microbial carbon sequestration efficiency and minimizing organic carbon mineralization loss. Consequently, SOC storage is higher in low-salt conditions compared to medium- and high-salt gradients, aligning with previous research findings (Liu et al., 2024).By the crop maturity stage, SOC content in the control group surpassed that in wheat cultivation plots. This indicates the dual impact of short-term carbon turnover during wheat cultivation (Li et al., 2018). At this stage, carbon input from wheat root exudates and residues decreases significantly, while grain development continues to deplete the soil’s active carbon pool. Microbial mineralization and decomposition of SOC persist, leading to SOC loss in wheat plots. In contrast, the control plots experience no crop-related carbon consumption.Under low-salt conditions, the soil aggregate structure remains stable, and salt moderately inhibits carbon-decomposing enzyme activity, allowing for the gradual accumulation of organic carbon. Observing the dynamic patterns throughout the growth period, it is evident that low-salt environments optimize the soil microenvironment, balancing organic carbon input and decomposition rates. This balance is advantageous for the long-term sequestration of organic carbon in saline-alkali soils (Guo et al., 2021; Liu et al., 2024).

During the jointing and flowering stages, DOC levels were lower than the control but surpassed it at the maturity stage, indicating a temporal interplay between carbon input and consumption rates. This aligns with LI Ling et al.’s (2013) findings that carbon source addition can elevate DOC content. However, most prior studies relied on short-term incubation experiments and did not capture the “first low, then high” trend throughout the entire growth period (Li et al., 2013). MBC accumulated progressively during the growth period, peaking under low-salt treatment at the flowering stage. This observation supports the salt threshold effect identified by LI Ling et al. (2013), where soil MBC was higher under low-salt (0.1%–0.5%) conditions and lowest under high-salt (0.9%) condition (Li et al., 2013). It also concurs with Boudabbous et al. (2022), who noted increased rhizosphere microbial activity at the flowering stage (Boudabbous et al., 2022). This study further integrates the salt effect with the growth-stage effect, revealing the synergistic impact of “low-salt + flowering stage” on MBC accumulation. ROC in wheat plots was generally lower than the control throughout the growth period, briefly surpassing it only at the flowering stage. This finding contrasts with ZHU Taochuan et al. (2022) and LI Chuanfu et al. (2023), who reported significant increases in ROC content due to fertilization (Zhu et al., 2022; Li et al., 2023). The discrepancy arises from differing research perspectives: while previous studies compared steady-state differences between fertilized and unfertilized treatments, this study highlights the dynamic effects of “root consumption” and “root input” during crop growth and non-growth conditions.Throughout most growth stages, microbial decomposition and root absorption rates exceeded root exudate input rates, with the carbon input rate only briefly surpassing the consumption rate at the flowering stage. Overall, the dynamic changes in DOC and ROC demonstrate that active organic carbon components in saline-alkali farmland are regulated by the balance between “carbon input” and “carbon consumption” rates. Meanwhile, MBC accumulation is driven by the salt threshold and peak growth period, together forming a multi-process response pattern of carbon turnover in saline-alkali land.

A significant positive correlation was observed among SAA, SOC, and ROC in wheat planting plots. This indicates that root exudates simultaneously supplement carbon and nitrogen substrates, stimulate enzyme-producing microorganisms, and facilitate the conversion of inert carbon to active carbon. These findings confirm the coupling relationship between carbon-nitrogen cycling and extracellular enzyme activity, aligning with field observations by Deng Xuzhe et al. (2023) and Ma et al. (2020). However, previous studies primarily focused on conditions without salt stress or on single straw returning patterns (Ma et al., 2020; Deng et al., 2023). This study elucidates the mediating role of SAA in carbon-nitrogen cycling under salt stress and highlights the unique regulatory characteristics of the coastal saline-alkali rice-wheat rotation system.

Conversely, a significant negative correlation was found between EC and ROC. High salinity inhibits the activity of mineralizing microorganisms, slowing the conversion of inert carbon to active carbon, consistent with conclusions from existing studies on saline-alkali soils (Deng et al., 2023). Additionally, a significant positive correlation between DOC and MBC was identified, as DOC serves as a readily available carbon substrate that directly promotes microbial biomass accumulation. However, AK, EC, and SCA were significantly negatively correlated with MBC, likely due to factors such as Na^+^ toxicity, ion competitive adsorption, and inhibition from intermediate metabolites produced during accelerated SCA decomposition. These variables exhibited a statistical correlation of synchronous decline without a unidirectional causal relationship. Furthermore, a significant negative correlation was noted between SCA and DOC. Cellulase decomposition products are easily adsorbed and fixed by colloids, reducing free DOC concentration. In the blank control group, a significant positive correlation among SOC, SMC, and ROC was observed, highlighting moisture as a crucial environmental factor regulating microbial activity and carbon conversion. AP and EC were significantly negatively correlated with SOC, as AP accumulation disrupts colloid adsorption stability and accelerates SOC loss. Meanwhile, AK enhances SAA activity, indirectly promoting DOC release.

Random forest analysis was utilized to quantify the independent predictive contributions of individual factors to SOC, while RDA was employed to elucidate the multifactor collaborative gradient differentiation patterns. Together, these models provided complementary insights into the driving mechanisms of the carbon cycle in saline-alkaline soils. Results from the random forest analysis revealed that, in wheat-planted plots, SSA made the most significant independent contribution to SOC. Wheat roots secreted sucrose-like substrates, which stimulated the proliferation of SAA. This proliferation accelerated carbohydrate decomposition, supplying substrates for microbial carbon sequestration, with both factors changing in tandem.In contrast, in the control plots without wheat, SCA and AP emerged as key predictors of SOC changes. Without exogenous crop carbon input, original cellulose-like residues in the soil were decomposed by SCA to supplement the carbon source. Excessive AP accumulation accelerated SOC decomposition through ion competition, with these factors fluctuating synchronously with SOC.RDA further clarified the multifactor collaborative gradient differentiation characteristics. In wheat-planted plots, SCA was identified as the core environmental factor regulating the gradient differentiation of active organic carbon components. The introduction of crop root stubbles and leaf residues enhanced SCA activity, dominating the spatial differentiation of active carbon components. Conversely, in the control plots, where there was no input of plant residues, the soil carbon pool primarily consisted of residual soluble sugars, making SSA the key factor in regulating active organic carbon differentiation.Comparative analysis with similar domestic and international studies on SOC dynamics in saline-alkaline farmlands indicated that most existing research focused separately on the effects of salinity gradients or single planting patterns on the carbon pool (Xu et al., 2026; Bai et al., 2026) Few studies have simultaneously coupled multidimensional factors such as water-salt, nutrients, and extracellular enzymes, or combined multiple statistical models to distinguish the independent contributions of single factors and multifactor collaborative gradient effects. Furthermore, existing studies have not systematically differentiated the mechanisms driving the carbon cycle in wheat-planting versus control systems. This study clarified that wheat planting reshapes the carbon cycle regulation network in saline-alkaline soils by altering substrate supply, thereby contributing to the differential regulation theory of the carbon cycle in coastal saline-alkaline rice-wheat rotation farmlands in the Yellow River Delta.

The PLS-PM path model elucidates the cascading regulatory dynamics of soil water-salt, nutrients, and extracellular enzymes on the organic carbon pool, overcoming the limitations of correlation analysis that cannot discern causal hierarchies.The global goodness-of-fit (GOF) values for the two models applied to wheat cultivation and the blank control were 0.42 and 0.39. The GOF values for both models meet the good-fitting standard of GOF ≥ 0.36 for heterogeneous soil datasets in the field (Tenenhaus et al., 2005; Wetzels et al., 2009; Tong et al., 2024). According to PLS-SEM methodological specifications, heterogeneous field ecological data require a multi-index joint evaluation system, including GOF, indicator loadings, path significance, and endogenous R. It is inadvisable to assess the model’s quality solely based on SRMR (Wetzels et al., 2009; Nguyen, 2025). The PLS-PM analysis results reveal that soil enzyme activity consistently emerges as the most stable core factor influencing soil organic carbon accumulation, whether in wheat-planted or bare land. The path “soil enzyme activity → total organic carbon” remains statistically significant even after 1, 000 Bootstrap resampling tests. This finding underscores the pivotal role of extracellular enzyme-mediated processes in organic matter decomposition and humification, which dominate soil carbon turnover and regulate organic carbon accumulation, even under saline-alkali stress conditions. This aligns with established principles of soil carbon cycling in coastal saline-alkali lands. Despite the limited statistical robustness of most direct skip-level paths, such as “water-salt → total organic carbon” and “nutrients → total organic carbon, “ due to the strong micro-scale spatial heterogeneity and patchy salt distribution in saline-alkali soils, the pure cascade chain model comparison reveals significant insights. When all direct skip-level effects are removed, the explanatory power for total organic carbon in bare land and wheat-planted groups drops by 97% and 67%, respectively. This dramatic decline confirms the ecological reality of a multi-path collaborative regulation pattern. Thus, the soil carbon cycling in saline-alkali lands cannot be fully explained by a simple step-by-step mechanism, and the limited robustness of individual paths does not negate their collective contribution to the overall regulatory framework. Moreover, the regulatory pattern exhibits clear habitat differentiation driven by vegetation cover. In bare land, abiotic processes primarily govern the carbon cycle. Here, water-salt conditions more strongly explain nutrient variation due to the upward migration of soil solutions, which enriches salt ions and mineral nutrients in the surface layer. The positive influence of soil water content on nutrient dissolution surpasses the inhibitory effects of salt stress, creating a positive correlation between water-salt conditions and nutrient content. Conversely, in wheat cultivation, the introduction of crop roots and the rhizosphere effect enhances organic substrate input, strengthening the coupling between nutrient supply, enzyme activity, and carbon pool transformation. This significantly boosts the model’s explanatory power for both active and total organic carbon, shifting the regulatory pattern from “abiotic stress-dominated” in bare land to a “synergy between abiotic factors and biological processes, “ thereby enhancing the stability and explanatory power of the carbon cycle regulatory framework.

## 5 Conclusions

This study systematically explored how wheat cultivation interacts with soil salinity gradients to influence soil environmental factors and the dynamics of the soil organic carbon pool in the coastal saline-alkali farmlands of the Yellow River Delta. It elucidated the distinct regulatory mechanisms of soil water and salt conditions, nutrients, and enzyme activities on organic carbon and its active components across various planting patterns. Additionally, the research identified the core regulatory pathways and characteristics of soil carbon cycling in these saline-alkali farmlands. The primary conclusions are as follows:

Throughout the growth period, wheat cultivation markedly altered the distribution of soil water, salt, nutrients, and enzyme activities in coastal saline-alkali land. Wheat transpiration and root water uptake notably decreased surface soil moisture. This reduction, coupled with soil evaporation and water-salt interactions, accelerated the upward movement of salt ions within the soil profile. Soil nutrients exhibited varied changes: in medium- and high-salt soils during later growth stages, phosphorus availability significantly declined due to crop uptake, whereas potassium levels remained relatively high, regulated by root activation and rhizosphere effects. Additionally, root exudates and organic residues significantly boosted the activities of soil hydrolases like sucrase and cellulase, enhancing the availability of metabolic substrates for soil microorganisms. Overall, across different salt gradients and growth stages, soil enzyme activities in wheat cultivation areas consistently surpassed those in bare control areas.

The interaction between salt gradients and wheat cultivation plays a crucial role in regulating the accumulation and turnover of soil organic carbon, particularly its active components. In low-salt environments, the toxic effects of salt ions on soil microorganisms are reduced. This alleviation, combined with the input of organic substrates from roots, enhances microbial metabolism and enzyme secretion. As a result, a balance is achieved between the input and decomposition of organic carbon, promoting the continuous accumulation of active carbon components such as dissolved organic carbon, microbial biomass carbon, and easily oxidizable organic carbon. In contrast, medium to high salt stress significantly inhibits microbial activity and enzyme efficiency, thereby weakening soil carbon turnover capacity and restricting the accumulation of the active carbon pool.

Wheat cultivation influences the turnover rate of soil active organic carbon, with its effect on total organic carbon stock being moderated by salt intensity. In soils with moderate to high salinity, wheat cultivation results in a reduction of surface organic carbon content over a short planting cycle, exhibiting a stage-specific pattern of “accelerated turnover and decreased stock.” Conversely, in low-salt soils, the carbon input from roots compensates for the carbon loss due to increased decomposition, resulting in no significant difference in total organic carbon content between wheat-planted areas and bare land.

In saline-alkali farmland, planting patterns significantly influence the core factors driving the organic carbon pool. In wheat planting systems, sucrase activity emerges as the primary factor affecting organic carbon variation. Conversely, in bare land systems, cellulase activity and available phosphorus content play more substantial roles. At the pathway level, extensive bootstrap resampling (1000 iterations) confirms that soil enzyme activity consistently serves as the most stable core factor for organic carbon accumulation across both systems. Comparative multi-model analysis reveals that wheat planting reconfigures the regulatory network sequence of “water-salt conditions → nutrients → enzyme activity → carbon pool” in saline-alkali land. This multi-pathway, coordinated regulatory approach significantly enhances the explanatory power of environmental factors on the variation of organic carbon components.

This study distinguishes between the immediate effects of wheat cultivation on surface carbon loss in saline-alkali soils and the long-term potential for optimizing the soil microenvironment. It elucidates the dual response mechanism of the carbon cycle in coastal saline-alkali farmland, considering both tillage and salinity gradients. By addressing gaps in existing research, it explores carbon dynamics during the growth period of saline-alkali soil under rice-wheat rotation and investigates the driving mechanisms behind these dynamics. The findings offer valuable scientific insights for selecting appropriate tillage patterns in the saline-alkali lands of the Yellow River Delta, maintaining soil carbon pools, and enhancing the ecological quality of saline-alkali soils.

This study primarily examined the short-term dynamic changes in surface soil during the growth period, neglecting the evolution of the deep surface soil carbon pool. It focused solely on the physical, chemical, and enzymatic properties of the soil, omitting crucial microscopic mechanisms such as soil microbial community structure and functional genes. Additionally, the experiment was limited to a single crop season, failing to capture the long-term evolution trends of the soil carbon pool under multi-year crop rotations.To address these limitations, future research should involve long-term fixed-position monitoring experiments, incorporating stratified sampling of profile soil. This approach would allow for a comprehensive exploration of the carbon pool’s evolution in saline-alkali soil profiles under extended rice-wheat rotation patterns. Furthermore, integrating high-throughput sequencing and metabolomics technologies could uncover the molecular mechanisms of soil microbial-driven carbon turnover under the combined effects of salt stress and crop cultivation. This would enhance the theoretical framework of carbon cycling in coastal saline-alkali farmland.

## Data Availability

The original contributions presented in the study are included in the article/**Supplementary Material**. Further inquiries can be directed to the corresponding author.
