# Supplementary material for: Dynamics of soil organic carbon under wheat cultivation in saline soil of the Yellow River Delta
Source: Front Plant Sci. 2026 Jul 31;17:1855435. doi: 10.3389/fpls.2026.1855435 (PMC13473129; doi:10.3389/fpls.2026.1855435)
Supplement: Supplementary file 1 [file DataSheet1.pdf]

Supplementary Table S1 Comparison of fitting indices of three competitive PLS-PM models

Note: WaterSalt is an exogenous latent variable with no R^2 value. The global GOF and SOC explanatory power of the pure cascading chain model are significantly lower than those of the baseline model, indicating that direct pathways skipping intermediate variables play an irreplaceable explanatory role. Although the parsimonious model has similar SOC explanatory power to the baseline model, it has a worse global fit and loses key intermediate regulatory pathways, which cannot meet the mechanistic analysis objective of this study.

Group	Model type	Global GOF	Nutrients R^2	Enzyme activity R^2	SOC active components R^2	SOC R^2
the blank control group	Baseline model (Figure 6)	0.3860	0.4315	0.2988	0.2244	0.6668
the blank control group	Pure cascading chain model	0.3080	0.6133	0.3229	0.0347	0.0209
the blank control group	Parsimonious model	0.3814	0.4206	0.2158	0.0444	0.6675
the wheat cultivation area	Baseline model (Figure 6)	0.4218	0.0977	0.2811	0.6137	0.7426
the wheat cultivation area	Pure cascading chain model	0.2715	0.1078	0.2526	0.0755	0.2437
the wheat cultivation area	Parsimonious model	0.3511	0.0876	0.2923	0.0896	0.7560

Supplementary Table S2 Path coefficients and robustness test results of the baseline model based on 1000-time bootstrap resampling

Note: * indicates that the 95% confidence interval does not cross zero, i.e., the pathway is statistically robust and significant. The core pathway "enzyme activity → total organic carbon" remains stably significant in both groups. The overall regulatory pattern is consistent with the baseline model (Figure 6), and the core conclusions of the model are robust and reliable.

（1）the blank control group

Pathway	Original coefficient	Resampled mean	Standard error	95% CI lower	95% CI upper	Robustness
water and salt → nutrients	0.6569	0.0341	0.5337	−0.7835	0.7603	Non-significant
water and salt → enzyme activity	−0.1225	−0.1434	0.3126	−0.6163	0.5182	Non-significant

Pathway	Original coefficient	Resampled mean	Standard error	95% CI lower	95% CI upper	Robustness
water and salt → SOC active components	−0.5101	−0.0333	0.4259	−0.8577	0.7585	Non-significant
water and salt → SOC	−0.5837	−0.1888	0.4749	−0.8051	0.7622	Non-significant
nutrients → enzyme activity	−0.4583	−0.2179	0.3629	−0.7379	0.5791	Non-significant
nutrients → SOC active components	0.5580	0.3226	0.5943	−0.8071	1.0122	Non-significant
nutrients → SOC	0.1170	0.0069	0.3931	−0.6320	0.7721	Non-significant
enzyme activity → SOC active components	−0.0996	−0.1989	0.3137	−0.7755	0.4960	Non-significant
enzyme activity → SOC	0.4597	0.4361	0.1753	0.1284	0.8107	Significant *
SOC active components → SOC	−0.1534	−0.1552	0.2393	−0.6137	0.3310	Non-significant

（2）the wheat cultivation area

Pathway	Original coefficient	Resampled mean	Standard error	95% CI lower	95% CI upper	Robustness
water and salt → nutrients	0.6569	0.0341	0.5337	−0.7835	0.7603	Non-significant
water and salt → enzyme activity	−0.1225	−0.1434	0.3126	−0.6163	0.5182	Non-significant
water and salt → SOC active components	−0.5101	−0.0333	0.4259	−0.8577	0.7585	Non-significant
water and salt	−0.5837	−0.1888	0.4749	−0.8051	0.7622	Non-significant

**Pathway**	**Original coefficient**	**Resampled mean**	**Standard error**	**95% CI lower**	**95% CI upper**	**Robustness**
→ SOC						
nutrients → enzyme activity	−0.4583	−0.2179	0.3629	−0.7379	0.5791	Non-significant
nutrients → SOC active components	0.5580	0.3226	0.5943	−0.8071	1.0122	Non-significant
nutrients → SOC	0.1170	0.0069	0.3931	−0.6320	0.7721	Non-significant
enzyme activity → SOC active components	−0.0996	−0.1989	0.3137	−0.7755	0.4960	Non-significant
enzyme activity → SOC	0.4597	0.4361	0.1753	0.1284	0.8107	Significant *
SOC active components → SOC	−0.1534	−0.1552	0.2393	−0.6137	0.3310	Non-significant
